# International Medical Congress

**Published:** 1886-01

**Authors:** 


### International Medical Congress.

As there seems to be quite an uncertainty in the minds of many, as to the success of the International Medical Congress, to be held in this country in 1887, we think it well to give the following editorial from the Medical and Surgical Reporter, of Philadelphia, of December 19. The statement is clear and unprejudiced. The desire in the Dental Profession is almost universal to have a Dental Section, if the Congress is to be a reasonable success. It is not strange that there are differences of opinion; all men do not agree upon any one subject. The idea that the Medical Profession is so barren of men of ability, that if a dozen, more or less, choose to stand outside, and even antagonize the interest of the Congress, that a sufficient number cannot be found in thirty thousand, to make a highly successful Congress, is, to say the least, not very complimentary to the profession.

" We have received a circular containing the preliminary organization of the International Congress, which we publish elsewhere, and which looks very much as though the meeting was destined to be a great success.

In this connection, it seems to us to be our plain duty to call attention to and to censure in very severe terms the unpatriotic course that has been hitherto pursued by the two prominent weekly medical journals, which, we are almost ashamed to say, have made every effort in their power to doom the Congress to failure.

With remarkable persistency, that would call only for the highest praise were it expended in a worthy direction, the *Medical Record*, of New York, and the *Medical News*, of Philadelphia, have, by repeated editorials and by the republication of extracts unfavorable to the Congress from interested foreign journals (such as those published in Berlin, which city was the great rival of the United States at Copenhagen for the honor of the Congress in 1887, and their utmost endeavors to cultivate the seeds of discontent that had been cast abroad by the disappointed clique. The editor of the *Medical News* is one of this select circle, and it is with sorrow that we witness his exhibition of weakness in prostituting the great mission of medical journalism, to the gratification of a jealous coterie. The *Medical Record* is the organ of the *New York circle*, and its great mission has been submerged by (to say the least) an exceedingly unpatriotic course.

How different in a similar situation would be, and indeed how dissimilar in the present case is the course pursued by the great, *independent* London weekly, the *Lancet*; this enormously influential journal, published in a country that would be perfectly excusable for hostility to an American Congress, has been much more loyal to our good name and our good fame than have our own journals. The scurrilous imputation has been made that the *Lancet* has been *bought* over, and as we write the words, we can see the smile that will illumine the face of Mr. Wakley when he reads that any one could for a momont suppose that his *absolute independence*, that has made his journal the most influential medical publication in the world, could be bought at any price. From the beginning, Mr. Wakley's penetration enabled him to recognize the true inwardness of this revolt, and rightly reasoning that justice, truth, and journalistic independence were to be placed high above all personal aims, he has accorded to the Congress a hearty support.

That our visitors will not see men worth seeing, and hear men worth hearing, because some of the leading lights of our large cities will "*sulk in their tents*," is not true. We venture to say that the distinguished physicians of Europe would consider it well worth their while to have traveled many miles to have heard and seen McDowell, Atlee, or Jenner; to see and hear Koch and many others whose names we could mention who did not, when they became famous, belong to *city cliques*. It is not always those who themselves think so, who are the most worth hearing and seeing.

Besides, we are not so sure that these *shining lights* will not be found at the feast. It is a significant fact, that the two journals already alluded to have, for some little time, dropped their former aggressive form and stand only on the defensive, editorially noticing the Congress only when their policy in the past is attacked, and then lamely defending their course, and endeavoring ludicrously to gracefully retire from a position which they evidently regret. Now, that those who would have taken pleasure in ruining the Congress, realize that the fœtus has great vitality and gives ample promise of a vigorous maturity, they hesitate in their unpatriotic course, and since success always assures success, commence to think that they would like to do homage to its shrine, and since, with a commendable spirit, the present authorities seem inclined to extend the hand of fellowship to those among the revolters whose co-operation is universally desirable, we doubt not that the chasm will be bridged, and that we will all hereafter

"Live in peace,
And die in a pot of grease."

In regard to the Dental Section, we append some extracts from an editorial in the British Journal of Dental Science, for November 15th; to this also, we ask special attention, as it shows the interest taken in the Section, on the other side, and we doubt not, the interest will increase, as the time for the Congress approaches."

"We have repeatedly drawn our readers' attention to the progress made, both upon this side and the American side, in the direction of forming an effective arrangement for the holding of this Congress. As was pointed out, there was at one time a most hopeless state of affairs. Internal, we might almost say petty personal, interests were introduced, which were wholly foreign to a congress of scientists, which should, above everything, be cosmopolitan. The section devoted to oral surgery and dentistry was, while these evil counsels prevailed, left out in the cold limbo of neglect. However, happier and wiser counsels have now won the day, and the section in which our profession takes the most interest has been reinstated. The vast importance of the International Congresses cannot be justly appreciated unless by those who have carefully attended the meetings, and assiduously *set themselves to learn.* That America is to be the next meeting place will, unless we are much mistaken, prove of considerable importance to us dentists. The conflict between English and American dentistry is still keen; we on this side have yet much to learn, and we stand an excellent chance of gaining the needed information, if we avail ourselves of this opportunity of going across the Atlantic rollers. The programme of that section has not at present reached us, but we have little doubt that it will be all it should be, and will afford everyone a chance of putting on record the results of his work and experience. The rules regulating the business of the Congress are fair, and open to only friendly criticism; we append the more salient ones for the perusal of our readers.

To urge upon English dentists " to be up and doing," will, we hope, be a work of supererogation. A man owes it alike to himself and his profession and country to take some part, even if it be only that of a hearer, in all such international meetings."

